# Effect of the hepatokine Tsukushi on HDL and reverse cholesterol transport

**DOI:** 10.1016/j.jlr.2026.101110

**Published:** 2026-07-20

**Authors:** Sum Lam, Ying Wong, Sammy Wing-Ming Shiu, Chi-Ho Lee, Kathryn Choon-Beng Tan

**Affiliations:** Department of Medicine, School of Clinical Medicine, The University of Hong Kong, Queen Mary Hospital, Hong Kong SAR, China

**Keywords:** Tsukushi, reverse cholesterol transport, cholesterol efflux capacity, high-density lipoprotein, apolipoprotein A-I

## Abstract

Tsukushi (TSK), a secreted protein of the leucine-rich proteoglycan family that functions as a hepatokine, is upregulated in liver diseases such as metabolic dysfunction-associated steatotic liver disease (MASLD). Clinical studies have shown that serum TSK levels inversely correlate with plasma HDL cholesterol (HDL-C) in MASLD. This study investigated the impact of hepatic TSK overexpression on reverse cholesterol transport (RCT) and HDL function. Golden Syrian hamsters were transduced with adeno-associated virus encoding TSK (AAV-TSK) and fed a standard chow diet. After 8 weeks, in vivo macrophage-to-feces RCT was assessed by intraperitoneal injection of tritium-labeled cholesterol-loaded macrophages, with serum, liver, and fecal samples collected at 48 h. Ex vivo HDL cholesterol efflux capacity was measured using RAW264.7 macrophages. Hepatic lipid metabolism gene expression, serum apolipoprotein A-I (apoA-I) levels, and lecithin-cholesterol acyltransferase (LCAT) and cholesteryl ester transfer protein (CETP) activities were quantified. TSK overexpression did not induce hepatic steatosis. At week 8, serum HDL-C and HDL-phospholipids were reduced (*P* < 0.05) in AAV-TSK hamsters compared with controls, whereas LCAT and CETP activities were similar. Ex vivo cholesterol efflux capacity of HDL was significantly diminished (*P* < 0.05). RCT was impaired, with tritium tracer reduced by 34.2% in HDL (*P* < 0.01) and 27.6% in liver (*P* < 0.05). Hepatic cholesterol content was reduced (*P* < 0.05), but triglyceride levels remained unchanged. Both hepatic and circulating apoA-I levels were significantly reduced (*P* < 0.05). TSK overexpression impaired RCT by suppressing apoA-I expression, reducing HDL-mediated cholesterol efflux, and thereby attenuating macrophage-to-feces cholesterol transport.

## Graphical abstract

**Figure:**
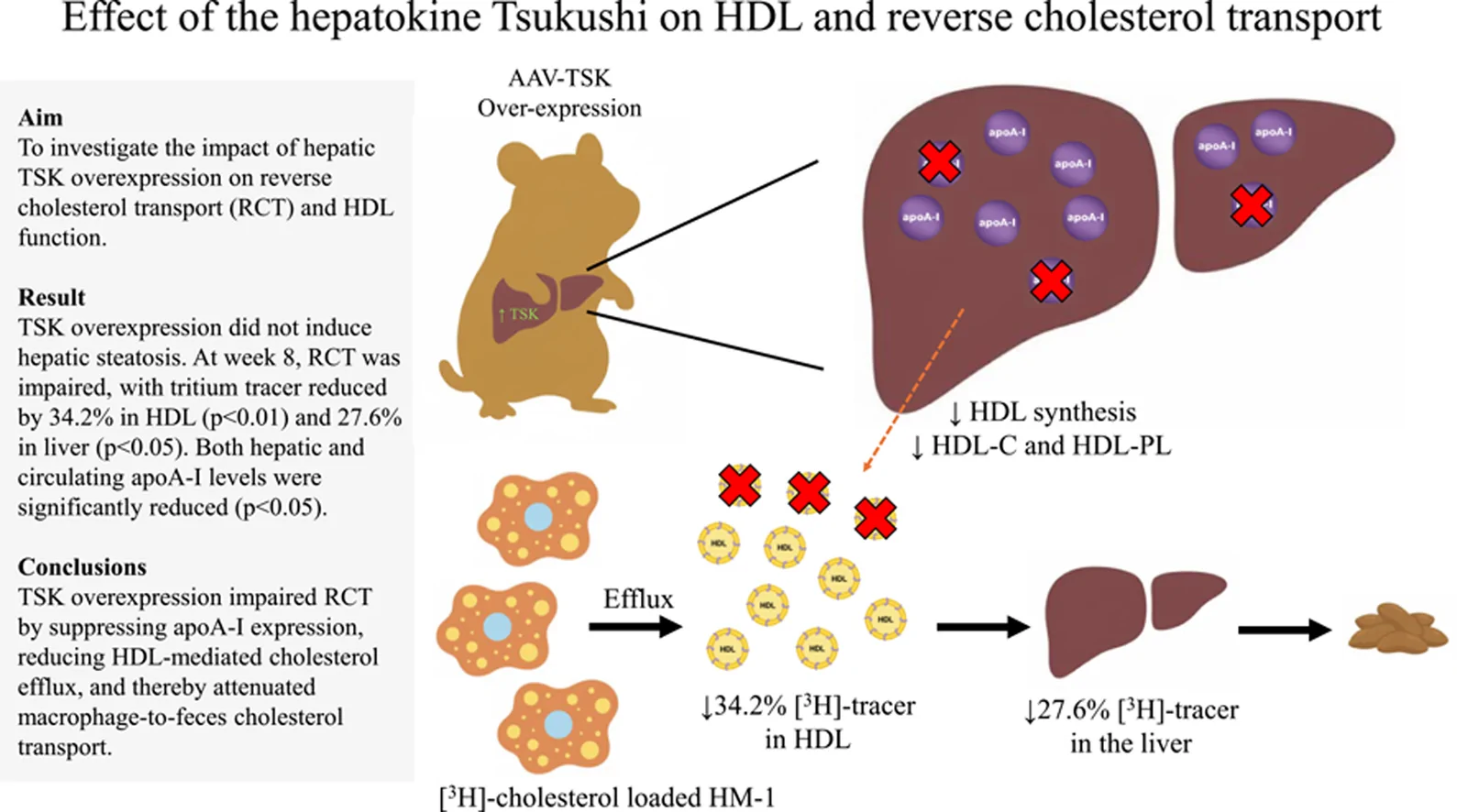

Tsukushi (TSK) is a small leucine-rich repeat proteoglycan (SLRP) that functions as a multifaceted extracellular signaling regulator, initially characterized for its role in developmental processes, especially during embryogenesis and organogenesis (1). More recently, it has been shown that TSK also functions as a hepatokine. Tsukushi is primarily produced and secreted by liver into circulation, and TSK expression was markedly increased in multiple mouse models of MASLD (2). In clinical settings, we and others have shown that patients with liver disease have increased TSK levels. Serum TSK levels were increased in subjects with MASLD and reflected the severity of liver fibrosis (3). In patients with acetaminophen-induced acute liver failure, elevated TSK level was associated with worse clinical outcomes (2).

Experimental studies have shown that TSK expression is induced in response to hepatic stress, with upregulation driven by endoplasmic reticulum stress and inflammation which are hallmark features of MASLD (2). It has been postulated that TSK is a serum biomarker of hepatic stress but whether TSK plays a role in glycolipid metabolism is unclear. Gain-of-function studies in mice have suggested that TSK can affect cholesterol homeostasis. Overexpression of TSK reduces circulating HDL cholesterol (HDL-C) and cholesterol efflux capacity (CEC) and decreases hepatic cholesterol-to-bile acid conversion (2). Since mice lack cholesteryl ester transfer protein (CETP) and have substantially higher HDL-C levels compared to humans, these cross-species differences limit the translatability of findings in murine studies in elucidating lipid metabolism to humans. CETP facilitates the transfer of cholesteryl esters from HDL to apolipoprotein B (ApoB)-containing lipoproteins (low-density lipoprotein (LDL) and very low-density lipoprotein (VLDL) and CETP is a crucial regulator of human lipoproteins remodeling and reverse cholesterol transport. HDL metabolism in mice is therefore different from that in humans and raises the possibility that the effect of TSK on HDL may not be the same in CETP-expressing species.

We have previously reported that serum TSK level inversely correlated with HDL-C and CEC in patients with metabolic dysfunction-associated fatty liver disease (MASLD) (4). Cholesterol efflux to HDL is the first step of reverse cholesterol transport (RCT) which removes excess cholesterol from peripheral tissues and returns to the liver for hepatobiliary excretion in feces. To elucidate whether there is a causal relationship between TSK and low HDL cholesterol, we proceeded to overexpress TSK in golden Syrian hamsters to determine the impact of TSK on HDL metabolism in the present study. Hamsters naturally express CETP at comparable levels to humans and exhibit HDL-C concentrations and lipoprotein profiles similar to those observed in humans (5). Hence, the hamster model provides a superior platform for studying lipid metabolism compared with mice. In addition, we intend to evaluate the impact of TSK overexpression not only on the ability of HDL to induce the first step of reverse cholesterol transport, but also on the entire RCT pathway. Our study would therefore comprehensively evaluate the role of TSK on the entire trajectory of cholesterol movement from peripheral macrophages through the HDL-mediated pathway to final fecal excretion.

## Materials and Methods

### Study protocol

Golden Syrian hamsters were transduced with either a TSK overexpressing AAV or a control AAV. Only 12-week-old male hamsters were used to ensure that the liver was fully grown and to avoid cyclic variations in hormones. Hamsters were housed in individually ventilated cages and maintained on a 12-h light and 12-h dark cycle at approximately 21–23°C. All hamsters were fed ad libitum a standard chow diet (SCD) (#5053, PicoLab Rodent Diet) for 8 weeks after transduction, and their diet consumption and body weights were recorded weekly. An in vivo macrophage-to-feces assay was performed to evaluate the reverse cholesterol transport pathway after 8 weeks of SCD feeding, and hamsters were euthanized thereafter, with the endpoint body weight and liver organ weight recorded. The study protocol was approved by the Committee on the Use of Live Animals in Teaching and Research, the University of Hong Kong.

### Liver-specific TSK overexpression in golden Syrian hamster

All golden Syrian hamsters were on a HsdHan®:AURA genetic background provided by the Centre for Comparative Medicine Research, the University of Hong Kong. The viral plasmid construction and virus packaging and purification service were provided by VectorBuilder. A TSK-overexpressing AAV plasmid (scAAV8-TBG-hamTSK) and a corresponding control AAV plasmid (scAAV8-TBG-NegORF) were designed to generate viral particles for the transductions of the experimental group (AAV-TSK) and the control group (AAV-CTRL), respectively. Self-complementary AAV of serotype 8 was chosen due to its superior liver tropism and better transduction efficiency derived from structural features of the AAV8 capsid (6, 7). Thyroxine-binding globulin (TBG) promoter was selected as a hepatocyte-specific promoter to enhance transgene expression preferentially to hepatocytes (8). Transductions were performed by intraperitoneal injection of viral suspension containing 2 × 10^12^ transduction units of either experimental or control AAV.

### Biochemical analyses of hamster serum, HDL fraction and hepatic extract

Hamsters were fasted for 6 h before measuring their blood glucose levels and collecting fasting serum samples for biochemical analyses at the baseline (week 0) and at the experimental endpoint (week 8) of the study. HDL fraction of fasting serum was isolated by precipitating out the ApoB-containing lipoproteins (LDL and VLDL) with precipitation buffer (#ab105138, Abcam), and the ApoB-depleted serum was used for subsequent biochemical measurements. Triglyceride, cholesterol and phospholipid in either serum or HDL fraction or the hepatic extract were quantified by commercial enzymatic colorimetric assays, including Stanbio™ Triglyceride (#2100-430, Stanbio Laboratory), LabAssay™ Cholesterol (#293-93601) and LabAssay™ Phospholipid (#295-94401, Wako Chemicals), respectively. Liver transaminases in serum were measured by commercial enzymatic colorimetric assays of alanine transferase (ALT) (#2920, Stanbio Laboratory) and aspartate transferase (AST) (#2930, Stanbio Laboratory). Serum lecithin-cholesterol acyltransferase (LCAT) and CETP activities were measured by commercial fluorometric assays. Serum LCAT activity assay (#RB-LCAT, Roar Biomedical) quantified the phospholipase activity of LCAT in catalyzing the transfer of a fluorescently labeled acyl group from the sn-2 position of phosphatidylcholine substrate to the 3-hydroxyl group of a cholesterol acceptor, resulting in the formation of cholesteryl ester. Serum CETP activity assay (#RB-CETP, Roar Biomedical) measured the transfer of fluorescently labeled neutral lipids from the synthetic lipoprotein-like donor particle to acceptor resulting in de-quenching of fluorophore.

### Hepatic lipid extraction

Hepatic total lipids were extracted according to the Folch extraction method (9). Briefly, liver tissue was homogenized and extracted with a mixture of chloroform and methanol. The lower phase was collected and evaporated, and the resulting lipid pellet was dissolved in a solvent mixture of isopropanol and NP40 and kept for subsequent measurements (10).

### Total RNA extraction and quantitative gene expression analysis

Tissues harvested from euthanized hamsters were preserved in RNAlater solution (#AM7020, Invitrogen) overnight at 4°C. Tissue samples were homogenized, and total RNA was extracted and purified. Standard complementary DNA synthesis and quantitative gene expression analysis was conducted according to the manufacturer’s protocol. Primer sequences are provided in Supplemental Table S1. Data analysis was performed using the fit-point method with a fluorescence threshold cycle (Ct) set at 2.5 arbitrary units (AU). Relative gene expression levels were normalized to hypoxanthine-guanine phosphoribosyltransferase 1 (*Hprt1*) as the reference gene.

### Protein extraction and quantitative protein expression analysis

Tissues collected from euthanized hamsters were homogenized in T-PER solution (#78510, Thermo Scientific). Protein expressions were quantified by standard western blotting technique. Protein expression levels of TSK, apolipoprotein A-I (apoA-I), CETP and beta-actin in serum or hepatic tissue were examined. Antibody details are provided in Supplemental Table S2. The specificity of anti-TSK and anti-apoA-I antibodies was confirmed using recombinant TSK and purified apoA-I protein as positive controls, respectively.

### Histochemical staining of formalin-fixed, paraffin-embedded hepatic tissue

Liver tissue histology and collagen deposition were assessed using Hematoxylin and Eosin (H&E) and Picrosirius Red staining, respectively. Hepatic tissue samples underwent standard histological processing and staining. Tissue sections were examined and digitally captured using a Nikon Ti2-E inverted microscope provided by the CPOS Imaging and Flow Cytometry Core facility, the University of Hong Kong.

### Cell culture

RAW264.7 cells, a murine macrophage-like cell line, were obtained from ATCC cell bank (#TIB-71). RAW264.7 cells were cultured in Dulbecco's Modified Eagle Medium (DMEM) (#11965092, Invitrogen) containing 10% fetal bovine serum (FBS) (#10270106, Invitrogen) and 1% Penicillin-Streptomycin (Pen/Strep) antibiotics (#15140122, Gibco). Cultures were kept in a humidified incubator maintained with 37°C and 5% CO_2_ environment. RAW264.7 cells were subcultured three times weekly at a split ratio of 1:8.

Macrophage-1 (HM-1) cell line is a macrophage line originating from lymphoid tumors of golden Syrian hamsters purchased from the JCRB cell bank (#JCRB1208, Osaka, Japan) (11). Cells were cultured in Roswell Park Memorial Institute (RPMI) medium (#11875093, Invitrogen) containing 10% heat-inactivated FBS (#A5256801, Gibco) and 1% Pen/Strep. Cultures were maintained at 37°C in a humidified, 5% CO_2_ environment with orbital shaking at 130 rpm (12). Cell cultures were partially refreshed twice weekly and kept within a density range of 2 × 10^5^ to 1 × 10^6^ cells/ml.

### Cholesterol efflux capacity of HDL ex vivo

The cholesterol efflux capacity (CEC) of hamster HDL was evaluated using a cell-based assay (13, 14). In brief, RAW264.7 cells were incubated with 0.6uCi/ml tritiated cholesterol ([1,2-^3^H(N)]-cholesterol) (#NET139005MC, PerkinElmer) for 48 h. After 48 h, the old medium was replaced with a fresh serum-free DMEM with 0.5% bovine serum albumin and 0.3 mM cyclic adenosine monophosphate (#B7880, Sigma, St. Louis, MO, USA) to upregulate ABCA1 transporter expression (15) and equilibrate cellular cholesterol pools for 20 h. On the last day, hamster apoB-depleted serum was prepared by mixing one part of the serum with one part of the precipitation buffer (#ab105138, Abcam, Cambridge, UK) to precipitate apoB-containing lipoproteins, and tritiated cholesterol-labeled RAW264.7 cells were then incubated with the supernatant containing the HDL fraction. HDL particles serve as extracellular acceptors to allow the efflux of tritiated cholesterol from RAW264.7 cells. Medium and cells were collected separately and quantified using a Tri-carb 4810TR liquid scintillation counter from PerkinElmer (MA, USA). The CEC of HDL was calculated as the radioactivity in media against the total radioactivity in both media and cells and presented in percentage.

### Macrophage-to-feces reverse cholesterol transport in vivo

Systemic reverse cholesterol transport pathway in hamsters was evaluated by an in vivo macrophage-to-feces assay. The principle is to intraperitoneally inject tritiated cholesterol-loaded macrophages (HM-1 cells) into recipient hamsters, and the presence of tritium tracer was tracked in different compartments, including serum, HDL fraction, liver, and feces after 48 h. HM-1 cells were incubated with fresh RPMI medium containing 1% FBS, 1% Pen/Strep and 5uCi/ml tritium labelled cholesterol for 48 h. Upon finishing the incubation, cells were washed and resuspended in Minimum Essential Medium (#42360032, Invitrogen, MA, USA). 1e7 cells carrying approximately 3.8e7 cpm radioactivity was injected into the peritoneal of recipient hamsters.

Total injected radioactivity and the presence of tritium tracers in whole serum and HDL fraction (ApoB-depleted serum) were counted directly with a scintillation counter. Tritiated cholesterol in the liver was extracted with the Folch extraction method (9) as previously described in the hepatic lipid extraction section with a minor modification in which the resulting lipid pellet was dissolved in toluene. The lipid solution in toluene was then added to scintillant and counted.

Tritiated cholesterol in the liver undergoes hepatic excretion into the intestine, and then appears in fecal matter, either remaining as neutral sterols or being metabolically transformed into bile acids. Given the distinct differences in polarity and solubility between these two lipid classes, a sequential two-stage solvent extraction procedure was employed to recover tritium tracers from both fractions. Briefly, fecal samples collected over a 48-h period were rehydrated and homogenized to obtain a uniform suspension. Neutral sterols extraction from fecal samples required alkaline saponification using ethanol and sodium hydroxide. The resulting unsaponifiable lipid fraction containing neutral sterols, specifically cholesterol, was then subsequently extracted with n-hexane to selectively extract these nonpolar lipids in the organic phase (16). The n-hexane fraction was evaporated, and the resulting lipid pellet was reconstituted in toluene. The remaining fecal mixture containing more polar lipids, including bile acids, was acidified by hydrochloric acid to protonate bile acids and render them extractable in organic solvent, prior to ethyl acetate extraction. The ethyl acetate fraction was evaporated, and the resulting bile acid-containing residue was reconstituted in isopropanol (17, 18). Both lipid solutions—toluene and isopropanol extracts, were combined with scintillation and quantified using a scintillation counter to determine tritium radioactivity. The percentage of radioactivity in each compartment was calculated as the ratio of tritium radioactivity in different compartments against total radioactivity injected.

### Statistical analysis

Data normality was assessed prior to statistical comparisons. Normally distributed data were analyzed using a two-tailed independent-samples *t* test and are presented as mean ± standard deviation, while non-normally distributed data were analyzed using the non-parametric Mann–Whitney *U* test and are presented as median and interquartile range. Statistical analysis and data visualization were conducted using Microsoft Excel (Microsoft) and Prism 10 (GraphPad), respectively.

## Result

### Hepatic TSK was overexpressed and serum TSK was elevated

**Fig. 1:**
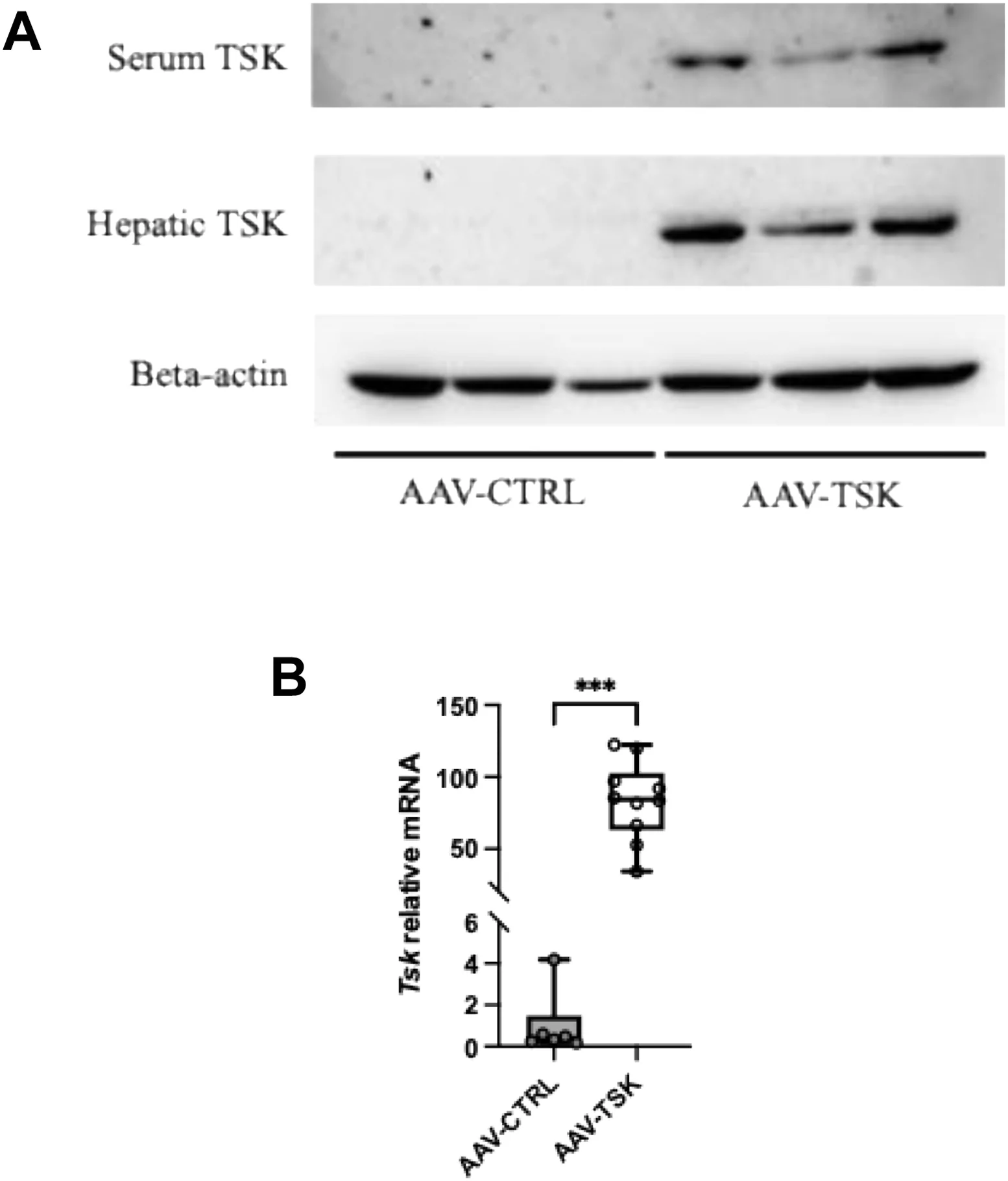
TSK Expression in AAV-Transduced Hamsters Figure 1. Serum and hepatic TSK expression in AAV-transduced golden Syrian hamsters at 8 weeks post-injection. A: Serum TSK, hepatic TSK and hepatic beta-actin protein expression. B: Hepatic *Tsk* mRNA expression. Values are expressed in median and interquartile range. AAV-CTRL, n = 6; AAV-TSK, n = 10. ∗∗∗*P* < 0.001 versus AAV-CTRL.

Hamsters were transduced with either a TSK overexpressing AAV or a control AAV. As shown in the western blotting analysis, the basal expression levels of serum and hepatic TSK were undetectable in the group of control hamsters whereas AAV-mediated overexpression of TSK resulted in significant upregulation of both hepatic and serum TSK protein levels (Fig. 1A). Hepatic *Tsk* mRNA expression was significantly increased in the AAV-TSK group as compared to AAV-CTRL group (Fig. 1B). These results showed that AAV transduction successfully enhanced the expression of endogenous TSK in the hamsters.

### TSK overexpression did not alter diet consumption, body weight as well as liver weight under SCD feeding

**Fig. 2:**
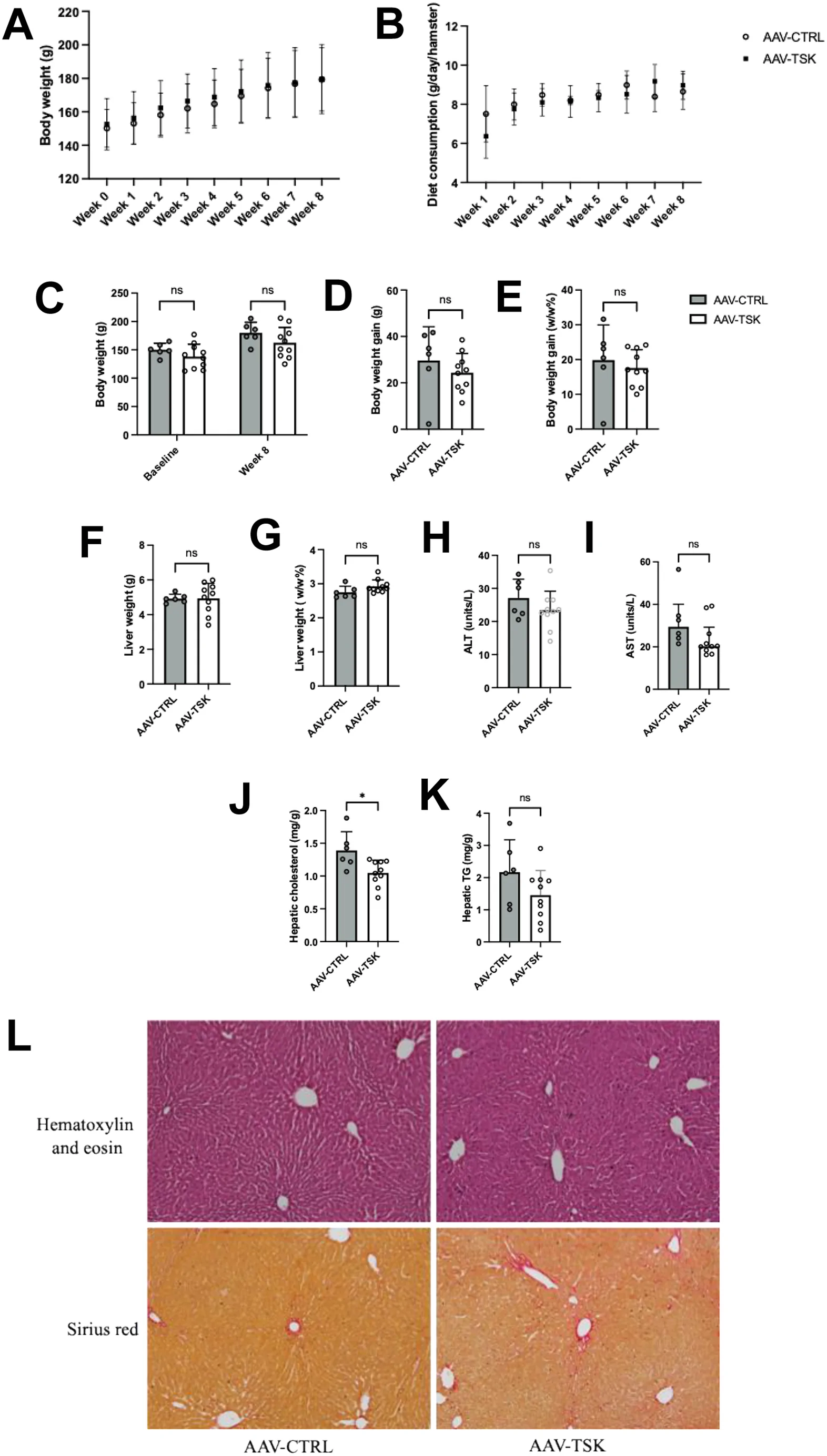
Body Weight, Hepatic Parameters, and Histological Analysis Figure 2. Metabolic and hepatic assessment of AAV-CTRL and AAV-TSK groups following 8 weeks of SCD feeding. A: Weekly body weight measurements. B: Weekly dietary consumption record. C: Body weight comparison at baseline and week 8. D: Absolute body weight gain over the 8-weeks observation period. E: Percentage change in body weight relative to baseline measurements. F: Liver weight at endpoint. G: Liver weight expressed as a percentage of total body weight. H: Serum ALT concentration. I: Serum AST concentration. J: Hepatic cholesterol concentration. K: Hepatic TG concentration. L: Representative histopathological images (10X) of liver tissue subjected to H&E staining and Picrosirius Red staining, demonstrating hepatic architecture and collagen deposition status. Representative images are presented for panel L. Values are expressed in mean ± standard deviation. AAV-CTRL, n = 6; AAV-TSK, n = 10. ∗*P* < 0.05 versus AAV-CTRL.

Longitudinal changes in body weight and dietary consumption are presented in Figure. 2A, B. No significant differences in body weight, cumulative body weight gain and liver weight were observed between AAV-CTRL and AAV-TSK groups at baseline or at week 8 (Fig. 2C–G). These results showed that AAV-mediated TSK overexpression did not substantially alter diet consumption, body weight as well as liver weight under SCD feeding conditions.

Serum liver transaminase levels also showed no significant differences between the two groups (Fig. 2H, I) suggesting AAV-mediated overexpression of TSK did not lead to hepatocellular injury. These findings were further supported by the histopathological examination. Interestingly, there was a reduction in hepatic cholesterol concentration in the AAV-TSK group compared to controls (*P* < 0.05) (Fig. 2J). Although the differences in hepatic triglyceride levels did not reach statistical significance (*P* = 0.13), a weak trend toward reduction was observed in the AAV-TSK group (Fig. 2K). Liver tissues from both AAV-CTRL and AAV-TSK groups demonstrated normal hepatic architecture with no signs of lipid accumulation, lymphocyte infiltration or collagen deposition, as assessed by H&E staining and Picrosirius Red staining (Fig. 2L). Collectively, these results indicated that TSK overexpression under SCD feeding did not elicit hepatic damage or fibrotic changes, except a modest reduction in hepatic cholesterol content.

### Reduced HDL-C and HDL-PL in the AAV-TSK group at week 8

**Fig. 3:**
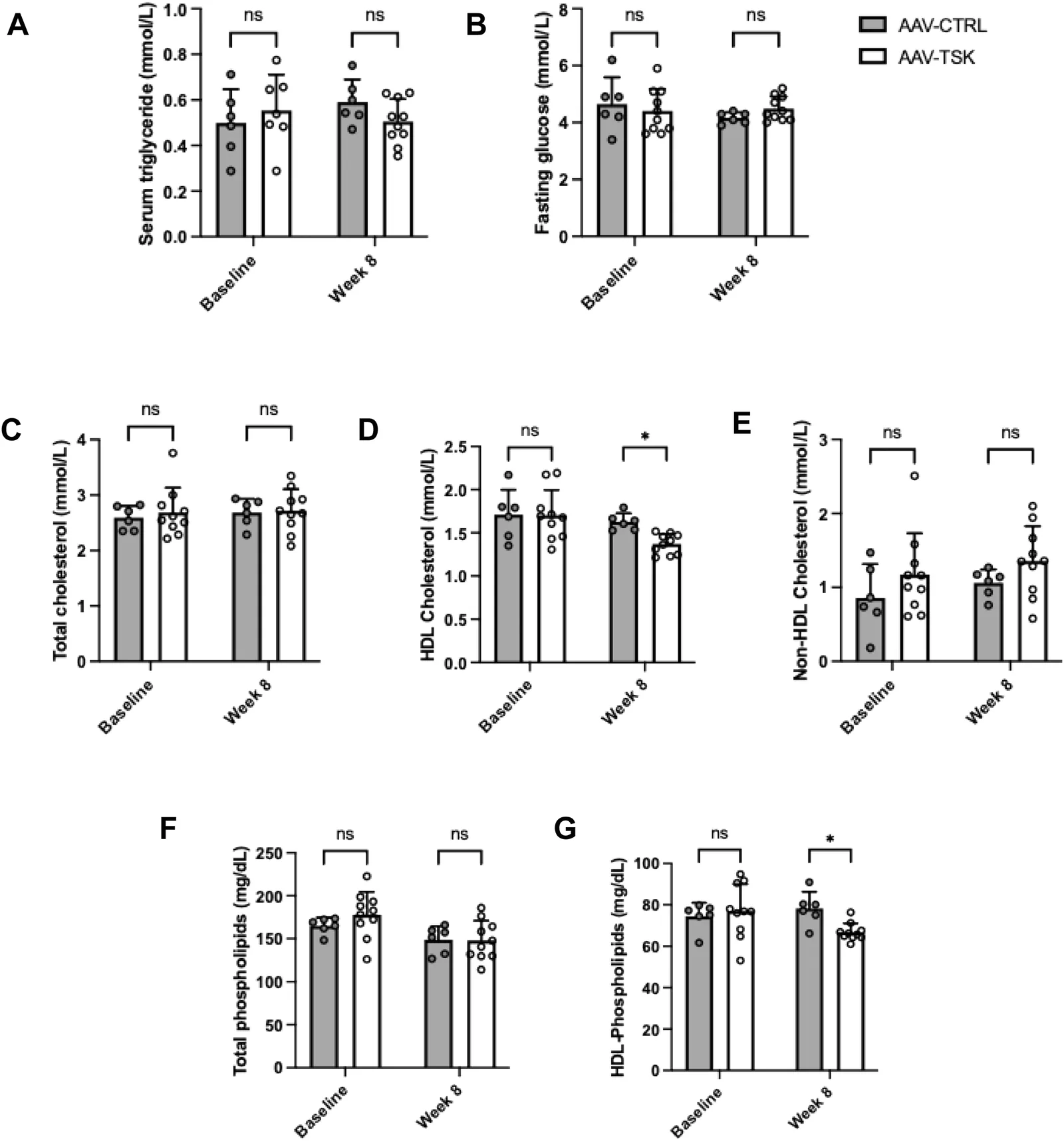
Serum Lipid and Glucose Profile in AAV-Transduced Hamsters Figure 3. Serum lipid and glucose profile of AAV-CTRL and AAV-TSK groups at baseline and week-8. A: Serum TG concentration. B: Fasting blood glucose levels. C: Total cholesterol concentration. D: HDL-C concentration. E: Non-HDL-C concentration. F: Total phospholipids concentration. G: HDL-phospholipids concentration. Values are expressed in mean ± standard deviation. AAV-CTRL, n = 6; AAV-TSK, n = 10. ∗*P* < 0.05 versus AAV-CTRL.

Serum lipid profile and fasting glucose levels at baseline were measured; overexpression of TSK did not affect serum lipid levels or fasting blood glucose levels (Fig. 3A–G). At week 8, both HDL-C and HDL-PL were reduced in the AAV-TSK group compared to control (*P* < 0.05) (Fig. 3D, G), whereas serum total cholesterol, non-HDL-C and total phospholipids remained comparable between groups (Fig. 3C, E, F). There were also no significant differences in serum triglyceride concentration or fasting blood glucose levels between AAV-CTRL and AAV-TSK groups at week 8 (Fig. 3A, B).

### TSK overexpression impaired macrophage-to-feces reverse cholesterol transport

**Fig. 4:**
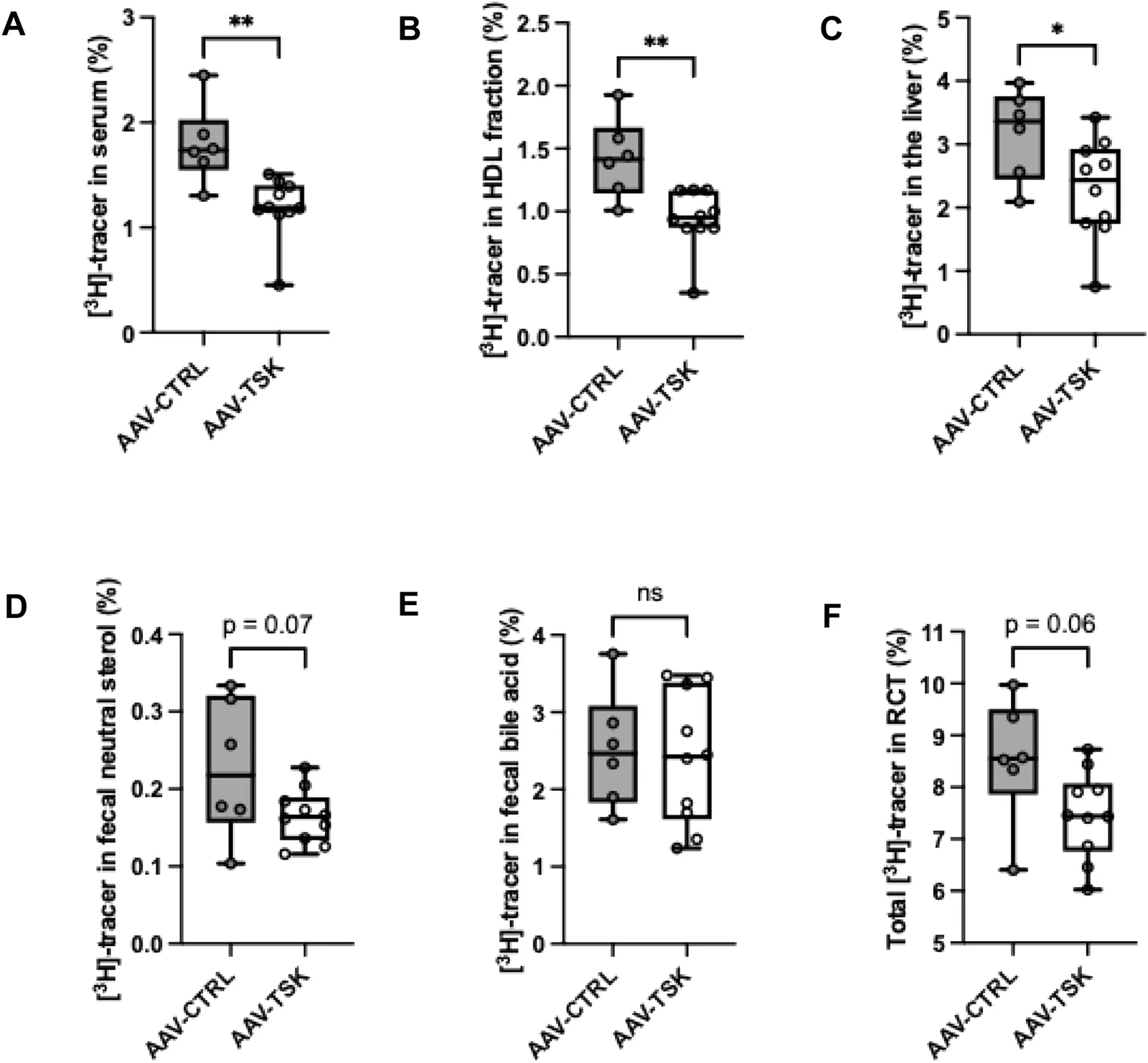
Reverse Cholesterol Transport In Vivo Figure 4. Assessments of macrophage-to-feces reverse cholesterol transport in vivo. A: [^3^H]-cholesterol tracer levels in serum. B: [^3^H]-cholesterol tracer levels in HDL fraction. C: [^3^H]-cholesterol tracer levels in the liver. D: [^3^H]-cholesterol tracer levels in fecal neutral sterol. E: [^3^H]-cholesterol tracer levels in fecal bile acid. F: Total [^3^H]-cholesterol tracer levels in RCT pathway. Values are expressed in median and interquartile range. AAV-CTRL, n = 6; AAV-TSK, n = 10. ∗*P* < 0.05, ∗∗*P* < 0.01 versus AAV-CTRL.

Since serum HDL was significantly lower in AAV-TSK hamsters, we further determined whether the overexpression of TSK would affect the whole RCT pathway by performing the in vivo macrophage-to-feces assay. After intraperitoneal injection of radiolabeled cholesterol-loaded macrophages, the appearance of tritium tracer in serum as well as in the HDL fraction were significantly reduced by 33.3% (*P* < 0.01) and 34.2% (*P* < 0.01), respectively, in the AAV-TSK hamsters (Fig. 4A, B). The amount of tritium tracer in the liver compartment was also 27.6% lower in AAV-TSK hamsters (*P* < 0.05) than control hamsters (Fig. 4C), suggesting reduced hepatic cholesterol uptake from HDL. In the final step of the RCT pathway, there was a trend towards a reduction in tritium tracer in fecal neutral sterols compartment (*P* = 0.07) in AAV-TSK hamsters (Fig. 4D) whereas no significant differences were seen in the fecal bile acid compartment (Fig. 4E). The total tritium tracer recovered from all compartments of the RCT pathway was reduced in AAV-TSK hamsters (Fig. 4F), albeit not statistically significant (*P* = 0.06). The data suggested that TSK overexpression caused impairments in the first and subsequent steps of RCT.

### Reduced cholesterol efflux capacity ex vivo in the AAV-TSK group

**Fig. 5:**
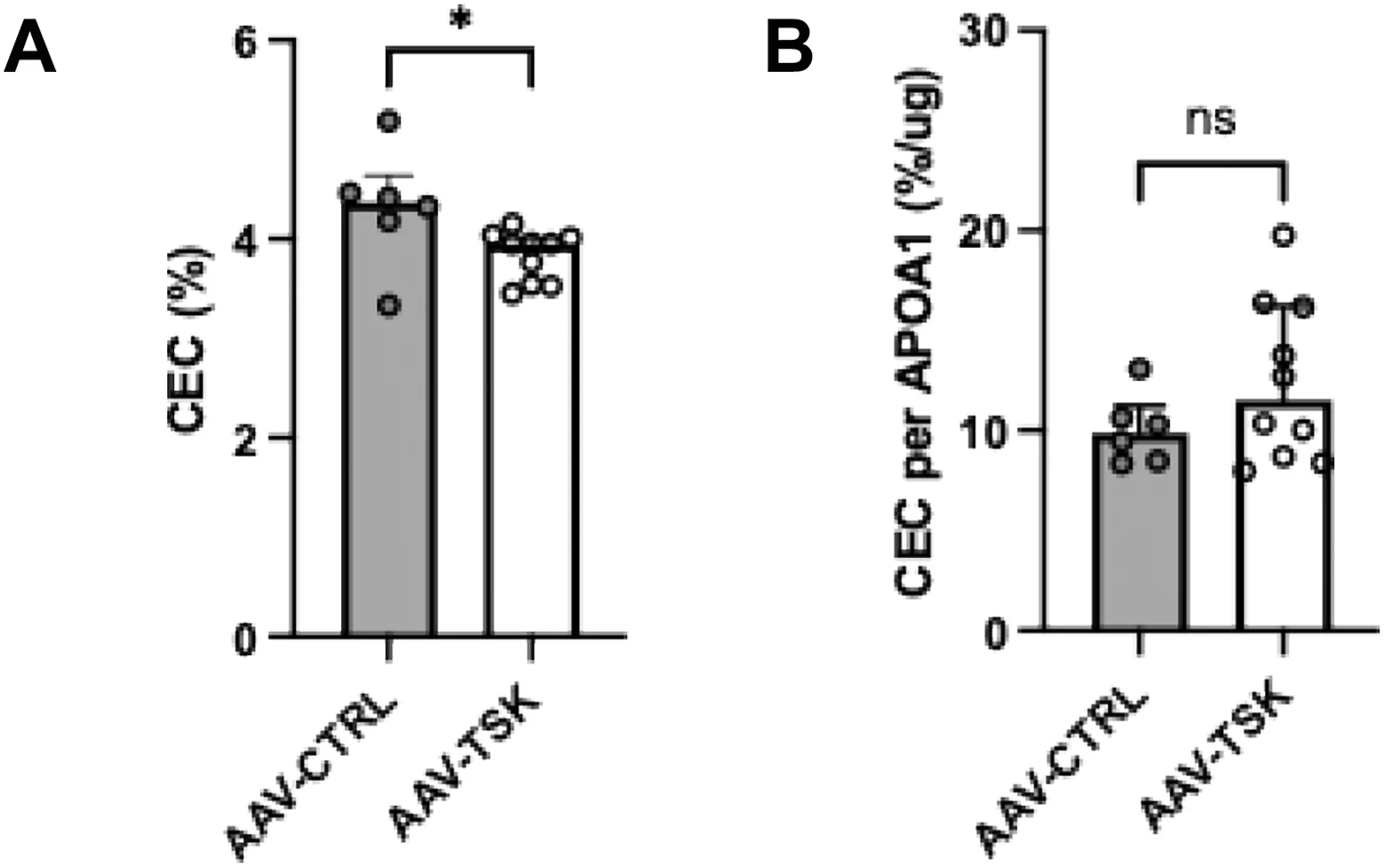
Cholesterol Efflux Capacity Ex Vivo Figure 5. Assessments of HDL cholesterol efflux capacity ex vivo. A: Cholesterol efflux capacity of HDL isolated from AAV-transduced hamsters. B: Cholesterol efflux capacity normalized against apoA-I concentration. Values are expressed in mean ± standard deviation. AAV-CTRL, n = 6; AAV-TSK, n = 10. ∗*P* < 0.05 versus AAV-CTRL.

The capacity of hamster HDL to induce cholesterol efflux from cholesterol-loaded RAW264.7 cells was also determined at week 8 ex vivo. When equal volume of apoB-depleted serum containing HDL was incubated with RAW264.7 cells, the total efflux capacity of HDL isolated from AAV-TSK hamsters was also significantly reduced compared to HDL from control hamsters (Fig. 5A). However, when we normalized CEC against serum apoA-I concentration, the differences were no longer significant (Fig. 5B). This would suggest that the reduction in CEC was mainly due to lower serum levels of HDL in AAV-TSK hamsters.

### TSK overexpression reduced apoA-I expression but did not affect LCAT or CETP activity

**Fig. 6:**
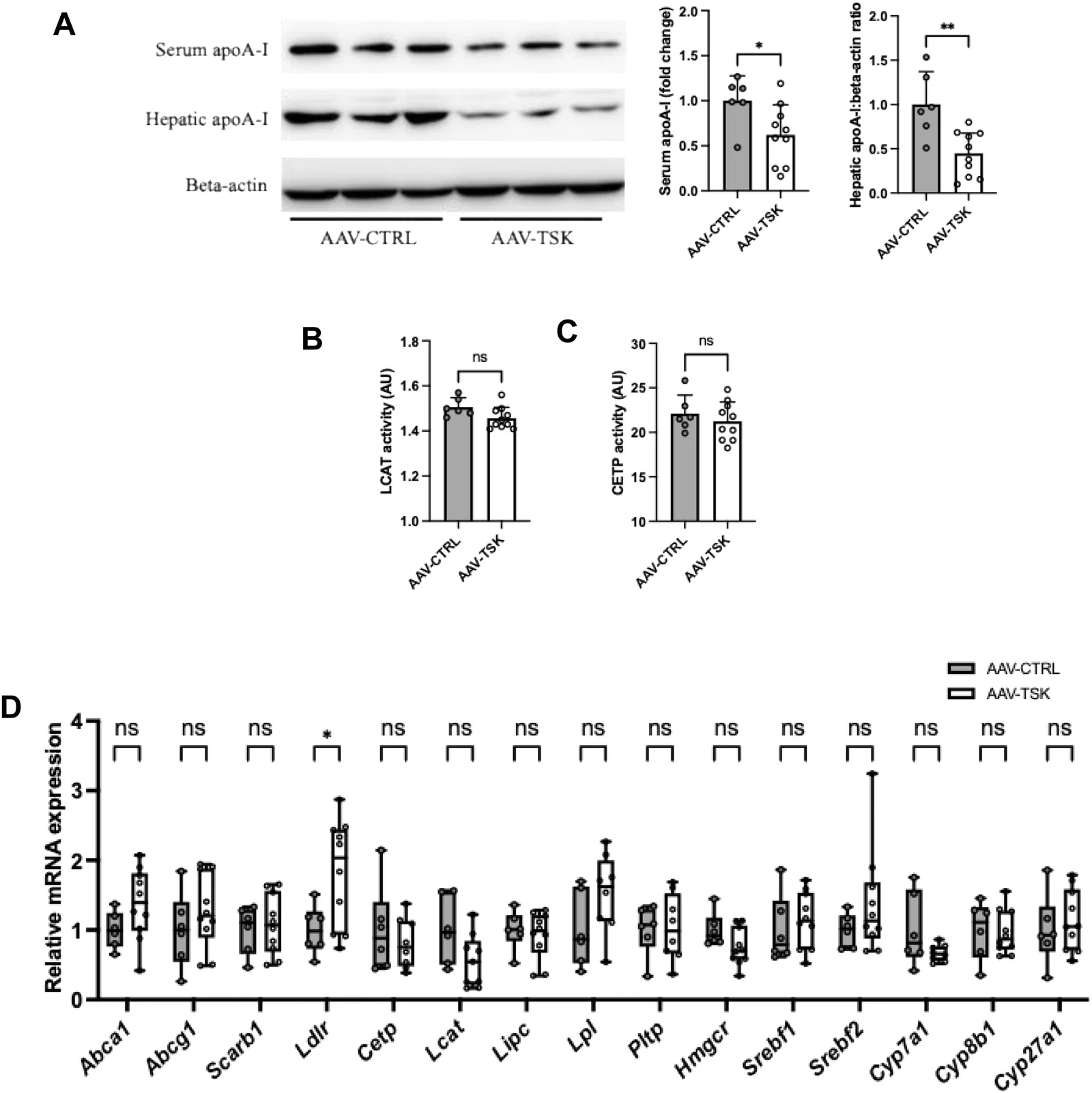
Cholesterol Metabolism Enzyme Activity and HDL Synthesis Assessments Figure 6. Assessments of the HDL synthesis by the liver and the enzyme activities related to cholesterol metabolism involved in HDL maturation and cholesterol transport. A: Protein expression levels of serum and hepatic apoA-I and hepatic beta-actin. B: Serum LCAT activity. C: Serum CETP activity. D: Relative mRNA expression levels of key receptors and enzymes related to lipid homeostasis. Values are expressed in mean ± standard deviation (A–C) and median and interquartile range (D). AAV-CTRL, n = 6; AAV-TSK, n = 10. ∗*P* < 0.05, ∗∗*P* < 0.01 versus AAV-CTRL.

To determine the effect of TSK overexpression on HDL synthesis, serum and hepatic apoA-I expressions were quantified. Western blotting analysis showed that hepatic apoA-I expression as well as the serum apoA-I levels were significantly reduced in the AAV-TSK group compared to controls (Fig. 6A). However, TSK overexpression did not affect enzymes involved in HDL maturation and cholesterol transport between lipoprotein particles, as there were no significant differences in both serum LCAT and CETP activities between two groups of hamsters (Fig. 6B, C). Serum CETP expression was also similar in the 2 groups of hamsters (Supplemental Fig. S1). Transcriptional analyses of hepatic tissue on key receptors and enzymes involved in lipid homeostasis revealed that most genes remained largely unchanged between the two groups, except for the upregulation of low-density lipoprotein receptor (*Ldlr*) expression which mediates cholesterol uptake from circulating LDL particles, in the AAV-TSK group compared to control (*P* < 0.05) (Fig. 6D). In contrast, scavenger receptor class B member 1 (*Scarb1*) expression, which facilitates hepatic cholesterol uptake from circulating HDL particles, remained unchanged between groups (Fig. 6D).

Pilot transcriptomic analysis was performed to investigate the potential mechanism(s) underlying the reduction in apoA-I expression in AAV-TSK hamsters. No changes were seen in the expression of genes known to regulate *Apoa1*, such as peroxisome proliferator-activated receptor alpha (*Pparα*) and hepatocyte nuclear factor 4 alpha (*Hnf4α*), suggesting that these canonical pathways are unlikely to account for the observed reduction. Interestingly, transforming growth factor β1 (*Tgf*-*β1*) was found to be upregulated in the liver of AAV-TSK hamsters. This finding was further validated by qPCR, which confirmed a 2.3-fold increase in *Tgf-β1* mRNA (*P* < 0.01) (Supplementary Fig. S2). Given that TGF-β1 has recently been shown to inhibit the transcription of *Apoa1* and *Apoa2* (19), the upregulation of TGF-β1 might be a plausible mechanism contributing to the lowered apoA-I expression observed in AAV-TSK hamsters.

## Discussion

This present study provided novel evidence that AAV-mediated TSK overexpression impaired reverse cholesterol transport and reduced apoA-I synthesis in golden Syrian hamsters fed on SCD. TSK overexpression did not induce the development of MASLD in our model and the observed abnormalities in RCT were therefore not a consequence of MASLD. Reverse cholesterol transport is the pathway that regulates cholesterol homeostasis by transporting excess cholesterol from extrahepatic tissues to nascent and mature HDL particles via ABCA1 and ABCG1 transporters. Free cholesterol acquired by HDL particles is esterified by LCAT and directly transported to the liver when mature HDL particles bind to hepatic SR-BI receptor which mediates selective cholesterol uptake by hepatocytes. Alternatively, cholesteryl ester in HDL particles can be transported back to the liver via an indirect route involving bidirectional lipid exchange. CETP mediates the transfer of cholesteryl ester from HDL to ApoB-containing lipoproteins (LDL and VLDL) in exchange for triglycerides, and these cholesteryl ester-enriched lipoprotein particles are then taken up by hepatocytes via binding to the LDL receptor and/or LDL receptor-related protein (20). Subsequent clearance of hepatic cholesterol is mainly through the biliary pathway and fecal excretion. Hepatic cholesterol can be directly excreted in bile or converted to bile acids by hepatic steroidogenic enzymes (CYP7A1, CYP8B1, and CYP27A1). Our findings demonstrated that AAV-mediated hepatic TSK overexpression significantly attenuated the initial steps of the RCT pathway. The reduced [^3^H]-cholesterol tracer accumulation in the HDL compartment suggested that there was an impairment in the capacity of HDL to stimulate cholesterol efflux from cholesterol-loaded macrophages in the AAV-TSK hamsters. This led to a decrease in cholesterol transport and uptake by the liver, as evidenced by the reduced [^3^H]-cholesterol tracer accumulation as well as cholesterol concentration in the liver. We also found a trend towards reduced tritium tracer in the fecal neutral sterol compartment in AAV-TSK hamsters, consistent with the observation that RCT was diminished and less cholesterol was excreted.

Our results suggest that the impairment in RCT in AAV-TSK hamsters is mainly caused by the decrease in circulating HDL to induce cholesterol efflux. The subsequent reduced hepatic cholesterol uptake from HDL was due to low HDL particles availability rather than alterations in hepatic SR-BI receptor expression as there were no significant changes in hepatic *Scarb1* expression. Our findings differed from those reported by Mouchiroud *et al*. who demonstrated that *Scarb1* expression was acutely reduced at 5-days post-transduction of AAV8-TSK but upregulated at 28-days post-transduction (2). These discrepancies likely reflect time-dependent regulatory adaptations and species-specific differences in metabolic regulation. While Mouchiroud *et al*. captured early transcriptional responses at 5-days and 28-days in mouse, we studied *Scarb1* expression at a much later time point (8 weeks after transduction) and *Scarb1* expression might have already reached a steady state and normalized. Interestingly, an upregulation of *Ldlr* expression was observed in our study. Since TSK overexpression resulted in a significant decrease in hepatic cholesterol content, this might activate negative feedback mechanisms through cholesterol-sensing mechanisms in hepatocytes, thereby increasing *Ldlr* transcription and subsequent LDL-dependent cholesterol uptake to compensate.

The cause of low HDL in AAV-TSK hamsters has been elucidated. We deliberately used an experimental model that would enable us to investigate the direct effect of TSK on HDL metabolism without the confounding effect of MASLD. It was clear that low circulating HDL in our AAV-TSK hamsters was not related to the metabolic effects of MASLD since none of the animals developed MASLD. Western blotting analysis revealed that both hepatic and circulating apoA-I levels were significantly reduced in AAV-TSK hamsters, whereas there were no significant changes in the activities of key HDL remodeling enzymes, including LCAT and CETP. Hence, TSK-mediated suppression of apoA-I synthesis was the main mechanism underlying the observed reduction in circulating HDL. These data extend previous reports demonstrating an inverse correlation between TSK and circulating HDL cholesterol levels in clinical studies (21, 22) and provide mechanistic insight into how TSK, as a negative regulator of HDL by the downregulation of apoA-I synthesis, diminishes the efficiency of RCT. Since TSK expression is induced in conditions like MASLD, we speculate that the resultant acute reduction of lipid influx may lessen further accumulation of lipids and ameliorate liver stress and damage. However, whether the sustained chronic increase in TSK in MASLD might potentially become “mal-adaptive” and contribute to the development of atherosclerosis by impairing RCT remains to be determined.

We have performed some preliminary experiments to investigate the molecular mechanism(s) on how TSK overexpression suppressed apoA-I levels. TSK has been reported to modulate multiple signaling pathways including Wnt, TGF-β1 and bone morphogenetic protein signaling in various cellular contexts (23), raising the possibility that TSK may interfere with transcriptional regulators of *Apoa1* through currently uncharacterized mechanisms. We have found that *Tgf-β1* was upregulated in the liver of the AAV-TSK group, and there is a recent study reported that over-expression of TGF-β1 inhibited the transcription of genes involved in cholesterol metabolism including *Apoa1* and *Apoa2* (19). We therefore hypothesize that TSK overexpression might upregulate hepatic TGF-β1 which in turn might suppress apoA-I expression. This hypothesis will need to be further tested in the future by determining the effect of inhibiting TGF-β1 on apoA-I in the AAV-TSK group.

The strength of our study is that we have used a hamster model to investigate the role of TSK in HDL metabolism and RCT, and our experimental design avoided any potential confounding effects caused by hepatic steatosis. There are several limitations that should be acknowledged. Firstly, while the intraperitoneal injection of radiolabeled cholesterol-loaded macrophages is the standard methodology for RCT assessment, we cannot completely exclude the possibility that a proportion of injected peritoneal macrophages might migrate to the liver during the experimental period. Second, our cholesterol efflux capacity assay employs single isotopic labeled cholesterol loaded macrophages as donor cells and apoB-depleted serum as the cholesterol acceptor. The assay mainly measures ABCA1-mediated cholesterol efflux to HDL because macrophages were pre-treated with cAMP to upregulate ABCA1 expression. We cannot address the bidirectional transport of cholesterol between HDL and cells as this would require double labeling of both cellular cholesterol and HDL cholesterol with different isotopes. Also, we did not perform investigations to specifically evaluate HDL function. Third, while the hamster model is superior for studying lipoprotein metabolism, it is not prone to atherosclerotic lesions development. Therefore, we cannot address whether impaired RCT and reduced HDL levels caused by TSK overexpression could lead to atherosclerosis. Additionally, the 8-week study design may not capture longer-term adaptive responses in lipid metabolism or potential cumulative effects of sustained TSK overexpression. Fourth, the findings of the present study observed in golden Syrian hamsters under standard chow-fed conditions cannot be extrapolated to metabolically stressed conditions such as high-fat diet feeding. Similarly, these findings may not be directly translatable to humans given inherent species differences in gene regulation, lipoprotein composition, and metabolic homeostasis. Finally, we have only performed preliminary experiments to elucidate the molecular mechanism(s) of how TSK overexpression might mediate the suppression of apoA-I levels and further mechanistic studies are warranted.

In conclusion, AAV-mediated TSK overexpression in golden Syrian hamsters did not induce hepatic steatosis but reduced HDL-C, CEC, and impaired RCT. This was mediated partly by a reduction in apoA-I synthesis.

## Data Availability

All the data supporting the findings of this study are presented in the manuscript and supplemental material.

## Supplemental data

This article contains supplemental data.

## Conflict of interest

The authors declare that they have no conflicts of interest with the contents of this article.
