# Supplementary material for: Effect of the hepatokine Tsukushi on HDL and reverse cholesterol transport
Source: J Lipid Res. 2026 Jul 20;67(8):101110. doi: 10.1016/j.jlr.2026.101110 (PMC13499385; doi:10.1016/j.jlr.2026.101110)
Supplement: Supplementary file 1 [file mmc1.docx]

**Supplementary Material**

**Effect of the hepatokine Tsukushi on HDL and reverse cholesterol transport**

Sum Lam^1^, PhD; Ying Wong^1^, BSc; Sammy Wing-Ming Shiu^1^, PhD; Chi-Ho Lee^1^, MD; Kathryn Choon-Beng Tan^1^, MD.

^1^Department of Medicine, School of Clinical Medicine, The University of Hong Kong, Queen Mary Hospital, Pokfulam Road, Hong Kong SAR, China.

**Supplementary Figure 1.**

Supplementary Figure 1. Protein expression levels of serum CETP. AAV-CTRL, n=6; AAV-TSK, n=10.

**Supplementary Figure 2.**

Supplementary Figure 2. Relative hepatic mRNA expression levels of *Tgf-β1*. Values are expressed in median and interquartile range. AAV-CTRL, n=6; AAV-TSK, n=10. **p<0.01 versus AAV-CTRL.

**Supplementary Table 1.**

| **Gene** | **Primer sequence (5’ 🡪 3’)** |
| --- | --- |
| *Tsk* | Fwd: TGGATGGGAACCCTCTGACT |
|  | Rev: AGCCCAGGTCTGAAAACACC |
|  |  |
| *Abca1* | Fwd: CCTCTGCTTGTGAGTGCTGGCC |
|  | Rev: GAAGACGAACACCACGCTGGGG |
|  |  |
| *Abcg1* | Fwd: TCCCTGTGGCCTACTGCAGCAT |
|  | Rev: GCGGCAAACAGCACAAATCGCA |
|  |  |
| *Scarb1* | Fwd: CCGCAGAATGTCCGCATAGA |
|  | Rev: TAGGACCTCGTTGGGGTTGA |
|  |  |
| *Ldlr* | Fwd: CACAGATCAACCCCCACTCC |
|  | Rev: GTGGGACATTGTCACGGACT |
|  |  |
| *Lcat* | Fwd: ATCACTGTCATCCTGTGCGG |
|  | Rev: AGAAAGTTTCCCTGGGGACG |
|  |  |
| *Cetp* | Fwd: GCTCCACCAGCACCTCCTACGA |
|  | Rev: GCCCTCTGGAAGGCTGTCTGGA |
|  |  |
| *Lipc* | Fwd: GGTCTCCCTGGCATACCAAC |
|  | Rev: CCAGGCTGTAGCCGATTAGG |
|  |  |
| *Lpl* | Fwd: ATACGGAGGTGGACATCGGA |
|  | Rev: GACACTTTCTCCCGAGCACA |
|  |  |
| *Pltp* | Fwd: GGACCTTCGCAGGTTTCAGA |
|  | Rev: CCAGGTCCGCTCATTAAGCA |
|  |  |
| *Hmgcr* | Fwd: CTGGTGGGTTCTGCCATGGCTG |
|  | Rev: TGTGCTGCATCCTGGCCACATG |
|  |  |
| *Srebf1* | Fwd: ACAGGACCTGAGCAGCCTGAGG |
|  | Rev: GCGGTCCAAGAGCTGGTGTGTC |
|  |  |
| *Srebf2* | Fwd: AGTGATCCGCCCACACTCACGA |
|  | Rev: CAGTGCCCGGCCCAAAACTGAT |
|  |  |
| *Cyp7a1* | Fwd: CTGCAAAGGCATTTGGACACA |
|  | Rev: TCCCTGGAGGGTCTTGGTAA |
|  |  |
| *Cyp8b1* | Fwd: GGAGTACCATATCCGCCGTG |
|  | Rev: GGGTTGAGGAACCGGTTGTA |
|  |  |
| *Cyp27a1* | Fwd: GCGCTTGCATGAACTACAGG |
|  | Rev: ATGGGCTTGAGTCCCAAAGG |
|  |  |
| *Hprt1* | Fwd: GCAGTCCCAGCGTCGTGATT |
|  | Rev: CCCCTTCAGCACACAGAGGG |

Supplementary Table 1. List of primer sequences used for qPCR analysis in golden Syrian hamsters.

**Supplementary Table 2.**

| **Protein target** | **Antibody** |
| --- | --- |
| Rat anti-TSK | #MAB6376, R&D System, MN, USA |
|  |  |
| Rabbit anti-apoA-I | #PA5-29557, Invitrogen, MA, USA |
|  |  |
| Mouse anti-CETP | #MA1-050, Invitrogen, MA, USA |
|  |  |
| Rabbit anti-beta actin conjugated with HRP | #5125, Cell Signaling Technology, MA, USA |
|  |  |
| Anti-rat IgG conjugated with HRP | #A5795, Sigma-Aldrich, MO, USA |
|  |  |
| Anti-mouse IgG conjugated with HRP | #7076, Cell Signaling Technology, MA, USA |
|  |  |
| Anti-rabbit IgG conjugated with HRP | #7074, Cell Signaling Technology, MA, USA |

Supplementary Table 2. List of antibodies used for detecting molecular targets in golden Syrian hamsters.
